# Characterization of Dimeric Vanadium Uptake and Species in Nafion™ and Novel Membranes from Vanadium Redox Flow Batteries Electrolytes

**DOI:** 10.3390/membranes11080576

**Published:** 2021-07-29

**Authors:** Christian Lutz, Michael Breuckmann, Sven Hampel, Martin Kreyenschmidt, Xi Ke, Sabine Beuermann, Katharina Schafner, Thomas Turek, Ulrich Kunz, Ana Guilherme Buzanich, Martin Radtke, Ursula E. A. Fittschen

**Affiliations:** 1 Institute of Inorganic and Analytical Chemistry, Clausthal University of Technology, Arnold-Sommerfeld Str. 4, 38678 Clausthal-Zellerfeld, Germany; christian.lutz@tu-clausthal.de (C.L.); sven.hampel@tu-clausthal.de (S.H.); 2 Department of Chemical Engineering, University of Applied Science Münster, Stegerwaldstr. 39, 48565 Steinfurt, Germany; michael.breuckmann@fh-muenster.de (M.B.); martin.kreyenschmidt@fh-muenster.de (M.K.); 3 Institute of Technical Chemistry, Clausthal University of Technology, Arnold-Sommerfeld Str. 4, 38678 Clausthal-Zellerfeld, Germany; xi.ke@outlook.de (X.K.); sabine.beuermann@tu-clausthal.de (S.B.); 4 Institute of Chemical and Electrochemical Process Engineering, Clausthal University of Technology, Leibnizstr. 17, 38678 Clausthal-Zellerfeld, Germany; katharina.schafner@alumni.tu-clausthal.de (K.S.); turek@icvt.tu-clausthal.de (T.T.); kunz@icvt.tu-clausthal.de (U.K.); 5 Forschungszentrum Energiespeichertechnologien, Am Stollen 19A, 38640 Goslar, Germany; 6 Federal Institute for Materials Research and Testing (BAM), Richard-Willstaetter-Str. 11, 12489 Berlin, Germany; ana.buzanich@bam.de (A.G.B.); martin.radtke@bam.de (M.R.)

**Keywords:** VRFB, PVDF-based membrane, UV/VIS, XANES, TXRF, ICP-OES, microXRF

## Abstract

A core component of energy storage systems like vanadium redox flow batteries (VRFB) is the polymer electrolyte membrane (PEM). In this work, the frequently used perfluorosulfonic-acid (PFSA) membrane Nafion™ 117 and a novel poly (vinylidene difluoride) (PVDF)-based membrane are investigated. A well-known problem in VRFBs is the vanadium permeation through the membrane. The consequence of this so-called vanadium crossover is a severe loss of capacity. For a better understanding of vanadium transport in membranes, the uptake of vanadium ions from electrolytes containing V_dimer_(IV–V) and for comparison also V(II), V(III), V(IV), and V(V) by both membranes was studied. UV/VIS spectroscopy, X-ray absorption near edge structure spectroscopy (XANES), total reflection X-ray fluorescence spectroscopy (TXRF), inductively coupled plasma optical emission spectrometry (ICP-OES), and micro X-ray fluorescence spectroscopy (microXRF) were used to determine the vanadium concentrations and the species inside the membrane. The results strongly support that V_dimer_(IV–V), a dimer formed from V(IV) and V(V), enters the nanoscopic water-body of Nafion™ 117 as such. This is interesting, because as of now, only the individual ions V(IV) and V(V) were considered to be transported through the membrane. Additionally, it was found that the V_dimer_(IV–V) dimer partly dissociates to the individual ions in the novel PVDF-based membrane. The V_dimer_(IV–V) dimer concentration in Nafion™ was determined and compared to those of the other species. After three days of equilibration time, the concentration of the dimer is the lowest compared to the monomeric vanadium species. The concentration of vanadium in terms of the relative uptake λ = n(V)/n(SO_3_) are as follows: V(II) [λ = 0.155] > V(III) [λ = 0.137] > V(IV) [λ = 0.124] > V(V) [λ = 0.053] > V_dimer_(IV–V) [λ = 0.039]. The results show that the V_dimer_(IV–V) dimer needs to be considered in addition to the other monomeric species to properly describe the transport of vanadium through Nafion™ in VRFBs.

## 1. Introduction

Renewable energy sources like wind, water, and solar power are sustainable alternatives to fossil fuels and nuclear energy. Their share in electrical power production is increasing constantly, e.g., in 2020 renewable energy sources produced 45.4% of the electrical power consumed in Germany [1]. Unfortunately, their production is usually less predictable in comparison to conventional power plants and thus not suitable for long-term power production. Energy storage systems are needed to store energy during times of high production and low demand [2,3,4]. Promising systems for stationary short- and long-term energy storage are redox flow batteries (RFB), which can theoretically provide unlimited capacity and possess a long lifetime of approx. ten years [5]. During recent decades, the vanadium redox flow battery (VRFB) has become one of the most advanced and most promising RFBs [6,7,8].

The VRFB consists of two half-cells, which are usually separated by an ionomeric membrane and connected to electrolyte tanks. During operation, the electrolytes are pumped through the half-cells. In the negative electrolyte (NE) V(II) is oxidized to V(III) during discharging and in the positive electrolyte (PE) V(V) is reduced to V(IV) [5,8].

The membrane in the VRFB affects the overall performance of the cell [9,10,11]. Due to its superior chemical/mechanical stability and good proton conductivity, the most frequently used membrane is Nafion™, a perfluorosulfonic-acid (PFSA). However, Nafion™ is expensive and has a poor [H^+^/V^n+^] ion selectivity [12,13,14]. In consequence, not only protons are transported through the membrane but also vanadium ions, often referred to as vanadium crossover. The transport of vanadium causes a concentration imbalance between the half-cells. According to the smaller size of V(II) and V(III) compared to the size of V(IV) and V(V), most authors report an increase in the total vanadium amount in the PE over several cycles. This phenomenon causes a self-discharge and a capacity fading of the battery [15,16,17,18,19,20,21,22,23,24,25,26], which requires adequate rebalancing strategies [27,28,29]. A better understanding of these phenomena is necessary to improve the performance of the polymer electrolyte membranes (PEM) in VRFBs and potentially also to improve PEMs in other applications.

Kusoglu and Weber reviewed the state of the art of molecular understanding on structure and transport in PFSAs, highlighting the complexity of water and proton uptake and transport [10]. Several studies show that water and proton uptake as well as transport is highly sensitive to environmental factors like temperature, acidity of the bathing solution, and membrane pretreatment [30,31,32,33,34,35]. The vanadium uptake and transport in PFSAs is even more complex. In the literature, different diffusion coefficients for the vanadium species are published, some deviating by orders of magnitude. For example, the published diffusion coefficients for V(III) are 7.12 × 10^−13^ [19], 5.93 × 10^−12^ [24], 3.22 × 10^−12^ [16], 1.87 × 10^−12^ [18], and 1.45 × 10^−11^ m^2^·s^−1^ [20]. Accordingly, it is not too surprising that models supposed to describe the transport phenomena are not consistent. Agar et al. modeled vanadium transport through Nafion™ based on the Nernst-Planck-equation including diffusion, migration, and convection [26]. However, Oh et al. modeled the vanadium transport through Nafion™ based on the Nernst-Planck-equation with negligible convection [17].

All in all, this indicates that vanadium crossover is not well understood and that the lack of knowledge is of a fundamental nature. For a better understanding of the vanadium crossover, it is necessary to determine vanadium concentrations and species in VRFB cell components, ideally in situ. In previous work, we have shown that redox reactions between vanadium ions can occur in the water-body of Nafion™ [36].

So far, the concentration of vanadium in Nafion™ has been determined by extraction. The group of Zawodzinski extracted vanadium from Nafion™ by immersing the membranes in nitric acid for 3 days [19,33]. The efficiency of the extraction procedure was not verified. Other methods for the vanadium determination in Nafion™ to the best of our knowledge have not been published so far.

The species determination of vanadium in electrolytes has been achieved ex situ using redox titration [37,38]. UV/VIS spectroscopy is often applied to study the vanadium species of the electrolyte in situ. Blanc et al. showed that in solutions with high concentrations of V(IV) and V(V) a strong absorbing dimer V_dimer_(IV–V) is formed [39]. The in situ determination of vanadium in NE using UV/VIS and linear combination was demonstrated in several studies, but due to the strong absorbing V_dimer_(IV–V) the speciation in the PE has rather high uncertainties [40,41,42,43,44,45,46]. Kausar et al. and Sun et al. have observed the formation of the dimer by Raman spectroscopy [47,48]. Lawton et al. showed that V(IV) can be determined in situ with electron paramagnetic resonance (EPR) [49,50]. Jia et al. determined the oxidation state of NE and PE in situ using synchrotron-based X-ray absorption near edge structure spectroscopy (XANES) [51]. In summary, the determination and speciation of vanadium in the electrolytes has been achieved by different independent methods. However, data on the vanadium species inside the nanoscopic water-body of the membranes are scarce. Vijayakumar et al. analyzed Nafion™ directly with UV/VIS and found only V(IV) inside Nafion™ after cycling [52]. To the best of our knowledge, this was the only approach to investigate the vanadium species in Nafion™ directly using UV/VIS.

The study presented here is contributing to the overall goal, which is to understand the chemistry of vanadium inside hydrated ionomeric membranes. Development of new procedures and methods for the determination and speciation of vanadium in situ are necessary to reach this goal. In this work, Nafion™ 117, a well-investigated PEM in VRFBs, and a novel membrane based on poly(vinylidene difluoride) (PVDF) were studied. The novel membrane is comparable to Nafion™ with respect to chemical/mechanical properties and proton conductivity but potentially more cost efficient and tunable with respect to better ion selectivity [53,54].

In this work, we focused especially on the vanadium dimer, which is formed from V(IV) and V(V) at high concentrations [39]. Until now, the dimer was not considered in transport models. It was thought to be unstable in the ionomers. We first determined the vanadium uptake from the V_dimer_(IV–V) dimer electrolytes. Subsequently, we identified the species inside the membrane to verify if the ions are still present as dimer or are dissociated into the individual ions. For comparison, other vanadium species V(II), V(III), V(IV), and V(V) were studied as well. Therefore, a procedure to determine the vanadium species inside the membranes by UV/VIS and XANES was established as well as a new procedure to extract vanadium from Nafion™ and other ionomeric membranes. The vanadium concentrations were determined using total reflection X-ray fluorescence spectroscopy (TXRF) and inductively coupled plasma optical emission spectrometry (ICP-OES). The extraction efficiency was validated by using micro X-ray fluorescence spectroscopy (microXRF).

## 2. Materials and Methods

### 2.1. Chemicals and Samples

#### 2.1.1. Materials

Sulfuric acid (concentrated), hydrogen peroxide (30%), and nitric acid (concentrated) were purchased from Merck (Darmstadt, Germany). Gallium standard (1 g·L^−1^) and vanadium standard (1 g·L^−1^) were purchased from Carl Roth (Karlsruhe, Germany). Ultrapure water was generated by Veolia Elga Purelab Flex 4 (conductivity: 0.055 µS·cm^−1^, Paris, France).

#### 2.1.2. Vanadium Electrolyte

Vanadium electrolytes were electrochemically converted from V(III/IV) electrolyte (vanadium concentration: 1.6 M, sulfuric acid concentration: 4 M, Gesellschaft für Elektrometallurgie mbH, Nürnberg, Germany) using an in-house VRFB cell described in [55]. The obtained vanadium species were evaluated using UV/VIS. The NE was analyzed using a 1 mm quartz cuvette (Hellma, Müllheim, Germany) and the PE employing a 0.1 mm flow through quartz cuvette from the same manufacturer.

#### 2.1.3. Membranes and Pretreatment

Nafion™ 117 from Chemours (thickness (dry membrane): 178 µm, equivalent weight: 1100 g·n(SO_3_)^−1^, Wilmington, DE, USA) and PVDF-based membrane (thickness (dry membrane): 150 µm) were used for the experiments. The PVDF-based membrane was prepared via graft copolymerization of 2-hydroxyethyl methacrylate (HEMA, Aldrich, St. Louis, MO, USA) and 2-acrylamido-2-methylpropane sulfonic acid (AMPS, Aldrich, St. Louis, MO, USA) on PVDF (Nowofol, Siegsdorf, Germany), which was activated by electron beam treatment with a dose of 200 kGy. The polymerization mixture consisted of 25 vol% monomer (mixture of 40 mol% HEMA and 60 mol% AMPS), 37.5 vol% water, and 37.5 vol% dimethyl formamide (DMF, ≥97%, Alfa Aesar, Kandel, Germany). At least ten minutes prior to heating of the reaction mixture purging with nitrogen was started to remove oxygen. After reaching the polymerization temperature of 70 °C, the activated PVDF base material was added to the reactor. After polymerization, the grafted PVDF was kept in a mixture of water and DMF overnight to remove residual monomer. Then, the material was washed with deionized water several times. The HEMA monomer units of the membrane were sulfonated with 2-sulfobenzoic acid anhydride (94%, Alfa Aesar, Kandel, Germany). For experimental details the reader is referred to references [53,54]. In the following, the PVDF-based membrane is abbreviated in the figures as PEM-N—the N stands for novel.

The membranes were cut into pieces with dimensions of 1 cm × 5 cm. The Nafion™ stripes were pretreated similar to Tang et al. [33]. Subsequently, Nafion™ was immersed in 3 wt% hydrogen peroxide, ultrapure water, 1 M sulfuric acid, and ultrapure water. Every step was performed for 1 h at 80 °C. Prior to this, the PVDF-based membranes were protonated with 1 M sulfuric acid for 24 h at room temperature.

#### 2.1.4. Conditioning and Extraction of Membranes

The pretreated Nafion™ cuts were dried in a drying oven for 1 h at 80 °C and 80 mbar, weighed, and submerged in either V(II), V(III), V(IV), V_dimer_(IV–V), and V(V) electrolyte (1.6 M in all cases) for 72 h at room temperature. The pretreated PVDF-based membranes were submerged without the drying step in either V(II), V(III), V(IV), V_dimer_(IV–V), and V(V) electrolyte (1.6 M in all cases) for 72 h at room temperature. The membranes immersed in V(II) were prepared and stored under a nitrogen atmosphere because of the high susceptibility of V(II) species to react with oxygen. Membranes conditioned with the different electrolytes were either studied directly with UV/VIS and XANES or subjected to the extraction process. For the extraction of the ions from the membranes water-body, the conditioned membranes were immersed in 15 mL 1 M nitric acid for 1 h at 100 °C. The membranes were removed from the bath and rinsed with ultrapure water. The bath and rinse solution were combined and ultrapure water was added to a total volume of 100 mL. The extracts were analyzed with ICP-OES and TXRF. Untreated membranes, membranes before extraction and membranes after extraction were analyzed with microXRF. For every species, six membranes were extracted.

### 2.2. Instruments

An analytical balance Satorius Entris (Göttingen, Germany) was used. A drying oven Binder VB23 (Tuttlingen, Germany) connected with a vacuum pump Edwards Vaccum E2M1.5 (Burgess Hill, United Kingdom) was employed to dry the samples.

For TXRF measurements, a Bruker Nano S4 T-Star was used (molybdenum tube, focusing multilayer monochromator, 50 kV, 1000 µA, 60 mm^2^ XFlash SDD, FWHM at Mn K_α_ < 149 eV, Berlin, Germany) and the software version 1.0.1.146. A gallium standard (198 µg·L^−1^) was used as the internal standard. A total of 10 µL of every sample was prepared on siliconized quartz-glass carriers and measured for 300 s.

The ICP-OES was an Agilent 5100 (Vertical Dual View, Dichroic Spectral Combiner, VistaChip II CCD detector, Santa Clara/CA, USA) equipped with a SeaSpray nebulizer (glass) and cyclonic spray chamber (glass). The spectra were corrected with fitted background correction.

A Bruker Nano M4 Tornado was used for microXRF (rhodium tube, polycapillary X-ray optic, 50 kV, 600 µA, 30 mm^2^ XFlash SDD, Berlin, Germany), the software version 1.6.0.286 and the plugin QMAP. For measurements, the membranes were placed on a polypropylene base plate. The spot size was 20 µm and the dwell time 20 ms·pixel^−1^. The stage speed for the untreated membrane and the membrane before extraction was 2.5 mm·s^−1^ and for the membrane after extraction 1.3 mm·s^−1^. For every membrane, an area of 40 mm^2^ was mapped. The pixel size for the untreated membrane and the membrane before the extraction was 50 µm and for the membrane after extraction 25 µm. All measurements were performed at 20 mbar (air atmosphere). Additionally, due to low counts in the membrane after extraction, nine pixels were summarized for analysis. The sum spectra were normalized on the associated measurements of the untreated membrane. For normalization, Rh L (2.63 keV to 2.93 keV) was used.

**Figure 1:**
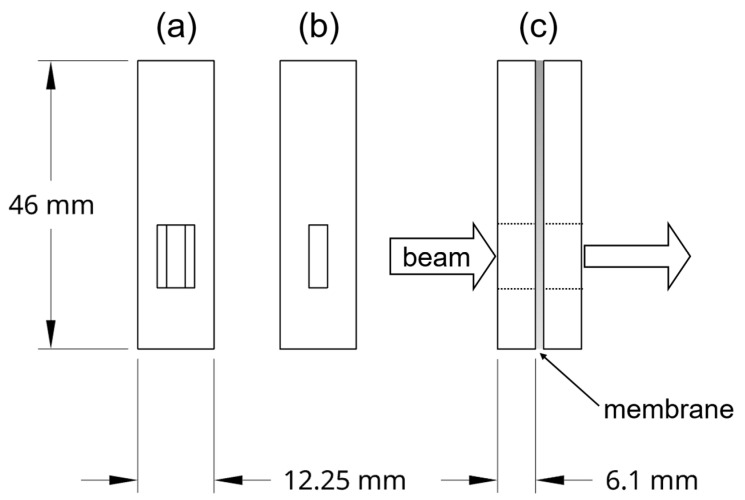
Sketches of (**a**) the outside and (**b**) the inside of the 3D printed cuvette for UV/VIS measurements of membranes, and (**c**) the application during measurements.

UV/VIS measurements were performed with a Jasco V-670 double beam spectrophotometer (Pfungstadt, Germany). For optimal positioning of the membranes inside the UV/VIS, 3D printed cuvettes were designed and printed using Innofil3D Pro1 (Emmen, The Netherlands) and an Ultimaker 3 3D printer (Geldermalsen, the Netherlands). In Figure 1, a sketch of the two parts of the cuvettes is shown. The membranes were put between the two blocks for the measurements. The faceted side of the window was to face outward and the straight cut side of the blocks had to face the membrane. The absorption between 300 nm and 900 nm of the membranes conditioned in the electrolyte was recorded. According to preliminary experiments, it is sufficient if the procedure was performed quickly for membranes immersed with V(II). Nafion™ was measured against air. The PVDF-based membrane was measured using a PVDF-based membrane hydrated with 4 M sulfuric acid as reference.

**Figure 2:**
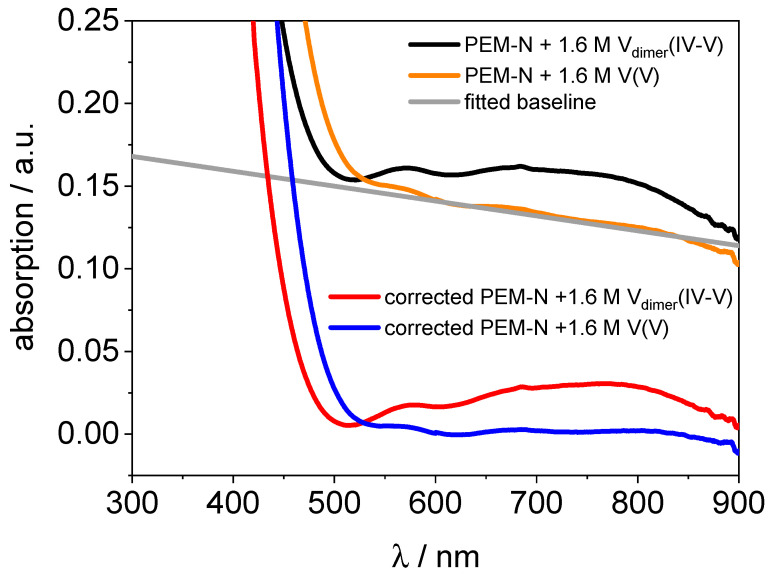
UV/VIS spectra of the PVDF-based membrane hydrated with 1.6 M V_dimer_(IV–V) and 1.6 M V(V) electrolyte and the baseline-corrected UV/VIS spectra are shown. A baseline, which was fitted to the UV/VIS spectrum of the PVDF-based membrane hydrated with 1.6 M V(V) electrolyte in the range 550 nm to 900 nm, is indicated.

The spectra of the PVDF-based membrane suffer from the high background absorption. Although the blank measurement of the membrane compensated some of the background, the spectra of the V_dimer_(IV–V) and V(V) remained significantly higher compared to the Nafion™ spectra. Due to the low concentrations of the species inside the membrane, the background absorption is considerably higher compared to the other species. Therefore, some spectra were additionally background corrected by the following procedure: The background was determined with the spectrum of the PVDF-based membrane hydrated with V(V). First, a baseline was fitted in the range of 550 nm to 900 nm. According to the UV/VIS spectra of the V(V) electrolyte and Nafion™ hydrated with V(V), the absorption in this range is nearly zero and can be used to fit the background. Then, the off-set and the slope of the baseline was determined, so that the spectrum of PVDF-based PEM hydrated with V(V) would match the typical spectra of V(V) determined in Nafion™ and the electrolyte. Last, the fitted baseline was removed from the original UV/VIS spectra and corrected spectra were obtained. In Figure 2, the background fit process and the corrected spectra of V(V) and V_dimer_(IV–V) in PVDF-based membranes are shown.

Laboratory-based XANES measurements in absorption mode were performed using an easyXES100 (EasyXAFS, Renton, WA, USA). The easyXES100 is equipped with a VF-80JM X-ray tube (W/Pd-anode, 4 mA, 25 kV, Varex Imaging, Salt Lake City, UT, USA) and a Ketek detector VITUS H80 80 mm^2^ SDD (Munich, Germany). The vanadium speciation was realized using a Ge(422) spherically bent crystal analyzer (SBCA). For every single spectrum, I_0_ was measured separately, without any sample in the beam path. The membranes were sealed between two polyimide foils (thickness: 40 µm, Conrad Electronic, Hirschau, Germany) for measurements. Every sample was measured ten times, the XANES spectra were collected, and merged afterwards for better statistics. The pre-edge and the edge region (5390 eV–5560 eV) were measured with energy steps of 0.25 eV and 4 s measurement time per data point. The post-edge region (5560 eV–5700 eV) was measured with 1 eV ΔE steps and 1 s measurement time per data point.

Synchrotron-based XANES measurements in fluorescence mode of both membrane types were performed at the BAMline (BESSY II, Berlin, Germany) [56]. The beam was monochromatized using a double Si (111)crystal monochromator (DCM) and an energy resolution of ΔE/E = 2 × 10^−4^. The incoming beam was monitored by a 5 cm long ionization chamber filled with air. The characteristic fluorescence radiation was measured with a custom made four-element SDD in backscatter geometry (LLA Instruments GmbH & Co. KG, Berlin, Germany). The single 30 mm^2^ detector modules were supplied by Ketek (Munich, Germany). The edge scan protocol was as follows: 5458 eV–5464 eV: ΔE = 3 eV, 5464 eV–5478 eV: ΔE = 0.5 eV, and 5478 eV–5600 eV: ΔE = 10 eV. The measurement time for every data point was 1 s.

As reference a V-foil (thickness: 5 µm, Exafs Materials, Danville, CA, USA) was used. For evaluation, the pre-edge peak (5466 eV–5474 eV, with an energy resolution of 0.5 eV) was fitted by linear combination. Data were normalized and evaluated using ATHENA [57].

## 3. Results and Discussion

Nafion™ and similar ionomers are sulfonic acid-based proton exchange membranes. After pretreatment, the sulfonyl groups of the membranes are considered to be fully protonated. Once immersed in the VRFB electrolyte the membranes are exposed to vanadium ions and additional protons. Though the proton content in the electrolyte of VRFBs is quite high (4 M sulfuric acid), protons are exchanged by vanadium ions inside the membrane and the concentration of vanadium in the membrane increases. It has been shown that vanadium ions like V(II), V(III), V(IV), and V(V) penetrate Nafion™ from aqueous solutions [16,18,19,50,58]. The relative uptake λ = n(V)/(SO_3_) were determined to be V(III) [λ ≈ 0.20], V(IV) [λ ≈ 0.19], and V(V) [λ ≈ 0.14] for a 1.5 M vanadium bathing solution [19]. It is well known that a V_dimer_(IV–V) dimer forms in the PE at states of charge (SOC) ranging from 10% to 90%. At 50% SOC almost only the dimer is present [42]. Interestingly, the dimeric species has not been considered to exist inside the membrane. It was generally assumed that individual ions V(IV) and V(V) enter the Nafion™ water-body at any SOC. However, if the dimer was stable inside the membrane it would have to be considered in transport models e.g., with its own diffusion coefficient. To shed light on this subject, the vanadium uptake from single species electrolyte and at PE with 50% SOC (dimeric vanadium) was determined as well as the vanadium species inside the membrane.

### 3.1. Vanadium Species Concentration Determination in Nafion™

**Figure 3:**
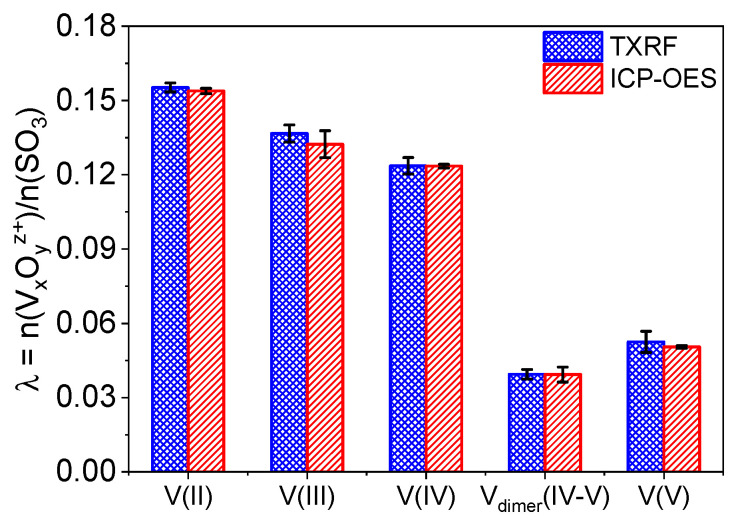
Uptake of V(II), V(III), V(IV), V_dimer_(IV–V), and V(V) (1.6 M in all cases) in Nafion™ using TXRF and ICP-OES (n = 6).

The concentration of vanadium inside the membrane is a measure of how receptible it is for the respective species and on how easily it can enter the water network. In Figure 3, the uptake of 1.6 M electrolyte containing either V(II), V(III), V(IV), V_dimer_(IV–V), or V(V) in Nafion™ determined by TXRF and ICP-OES is compared. The relative uptake λ was derived using the vanadium amount determined in the extract, the extraction volume, the dry weight of the individual piece of membrane, and the equivalent weight given by the manufacturer.

The V(II) ion shows the highest uptake with λ = 0.155 and the dimer V_dimer_(IV–V) the lowest with λ = 0.039. Vanadium(V) is the monomeric vanadium species with lowest uptake (λ = 0.053). According to literature, the uptake is governed by ion radius, Stokes radius, charge, and charge density [19,49]. The transport of large ions may be hindered due to the small size of the water channels of the membrane. The order of Stokes radii r_S_ for the monovalent vanadium ions is V(II) [r_S_ = 0.32 nm] = V(III) [r_S_ = 0.32 nm] > V(V) [r_S_ = 0.28 nm] > V(IV) [r_S_ = 0.21 nm] [59]. The Stokes radius of V_dimer_(IV–V) is unknown. Besides the size, the charge/ionic valance influences the uptake of the cation. Therefore, the Donnan potential has a higher effect on cations with a large charge and are more strongly repulsed by the membrane [60]. Both factors, size and charge, are combined in the charge density.

The Stokes radii and the charge allows to determine the z/r ratio for each ion, which results in the following ranking V(IV) > V(III) > V(II) > V(V). The uptake correlates with the reverse order of the z/r ratio of the ions besides V(V). It exhibits the lowest uptake of all monovalent ion species. However, it has been discussed that V(V) could be present as a polymeric form for sulfuric acid concentration < 7 M [61]. Therefore, the Stokes radius of the polymeric V(V) is significantly higher and thus the uptake is limited mainly by the ion size. It has been hypothesized that the affinity of the sulfonic acid in Nafion™ is higher to vanadium ions with lower oxidation state [60]. Since V_dimer_(IV–V) is the only divalent vanadium species, it has a significantly larger Stokes radius similar to the polymeric form of V(V). Therefore, the uptake of this ion should be also limited by the size and indeed it has the lowest uptake of all vanadium species present in vanadium electrolytes. Additionally, the uptake of V_dimer_(IV–V) is fundamentally different from an uptake represented by a linear combination of both individual V(IV) and V(V) species.

In summary, the concentrations of vanadium species found inside Nafion™ have the following order: V(II) > V(III) > V(IV) > V(V) > V_dimer_(IV–V). This is in accordance with the findings of Elgammal et al. [19] and Cho et al. [60], besides they did not study the dimer. However, it is surprising that the concentrations determined here are considerably smaller compared to those found by Elgammel et al.—the difference between the uptake values is in the order of λ = ~0.07. This may be explained by a different sample preparation procedure. In contrast to those studies, here an additional drying step was applied. Since it is known that the permeability decreases due to drying [62], the deviations may be explained.

**Figure 4:**
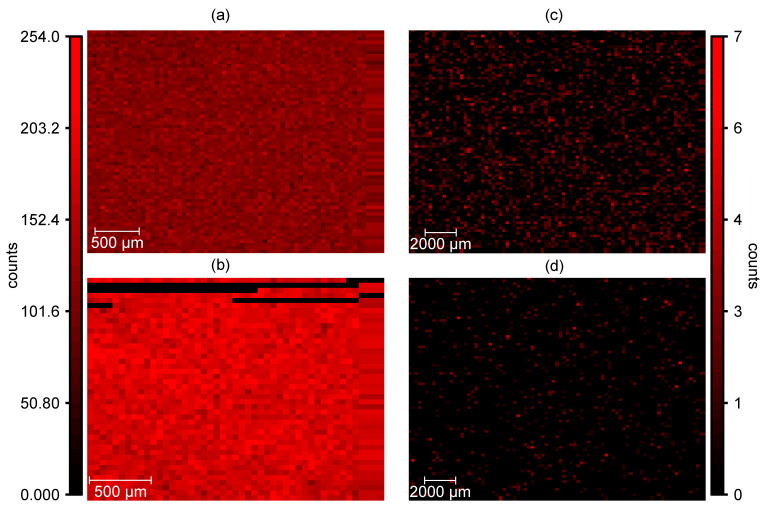
V(IV) distribution (counts per pixel) in (**a**) Nafion™ and (**b**) PVDF-based membrane before extraction, and in (**c**) Nafion™ and (**d**) PVDF-based membranes after extraction using microXRF.

Nonetheless, incomplete extraction could also result in lower concentrations. To verify the efficiency of the vanadium extraction, the vanadium distribution in Nafion™ and the PVDF-based membrane, both conditioned with V(IV) before and after extraction, were analyzed. The results are shown in Figure 4. Before extraction, V(IV) is distributed evenly in both membranes (s. Figure 4a,b). The low count artifacts in Figure 4b are presumably caused by a low hydration of the membrane in this area. After the extraction procedure, the vanadium fluorescence decreases significantly in both membranes (s. Figure 4c,d). Nevertheless, the concentration of vanadium remaining in Nafion™ after extraction is higher than in the PVDF-based membrane.

**Figure 5:**
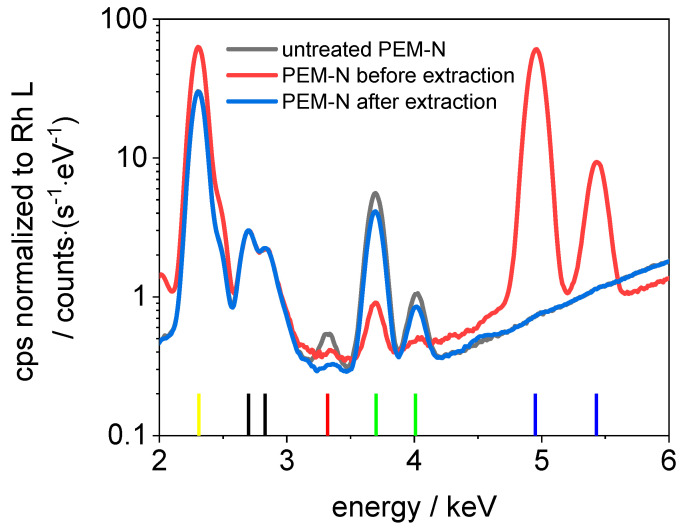
Sum spectra of untreated Nafion™, Nafion™ before and after extraction (normalized on Rh L). Vertical reference lines: yellow: S K_α_, black: Rh L_α_ and L_β_, red: K K_α_, green: Ca K_α_ and K_β_, and blue: V K_α_ and K_β_.

**Figure 6:**
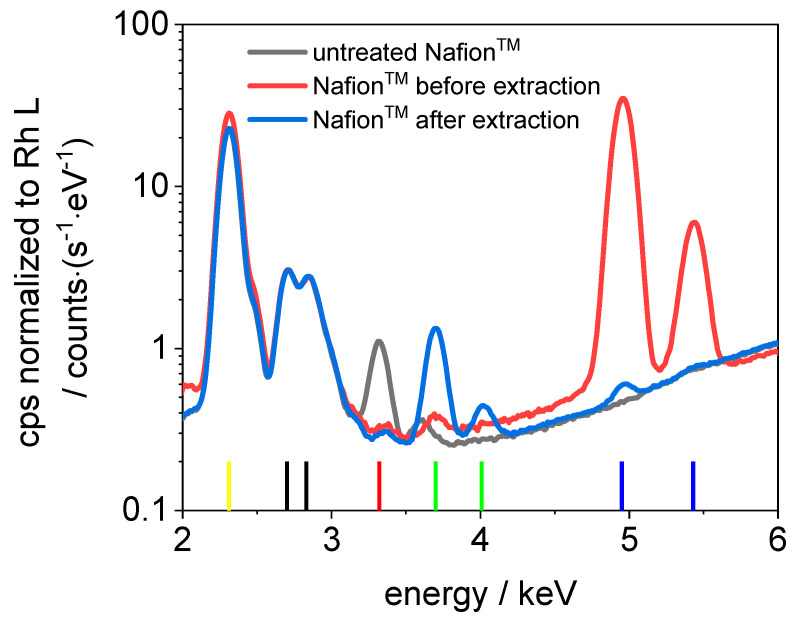
Sum spectra of untreated PVDF-based membrane, PVDF-based membrane before and after extraction (normalized on Rh L). Vertical reference lines: yellow: S K_α_, black: Rh L_α_ and L_β_, red: K K_α_, green: Ca K_α_ and K_β_, and blue: V K_α_ and K_β_.

In Figure 5 and Figure 6, sum spectra from the elemental maps in Figure 4 are shown, as well as sum spectra of an untreated Nafion™ and PVDF-based membrane. The following lines are present in all spectra: S K_α_ (2.30 keV), Rh L_α_ (2.70 keV), Rh L_β_ (2.83 keV), K K_α_ (3.32 keV), Ca K_α_ (3.69 keV), Ca K_β_ (4.01 keV), V K_α_ (4.95 keV), and V K_β_ (5.43 keV). The sulfur is an integral part of the sulfonic acid in Nafion™ and the PVDF-based membrane. The rhodium lines originate from scattering of the excitation radiation. A possible source of the potassium and calcium lines is the polypropylene base plate. Vanadium is not present in measurable concentrations in the untreated membranes. According to fundamental parameter-based calculations provided by the manufacturer’s software, the concentration of vanadium ions in Nafion™ after the extraction can be estimated to be <100 ppm (determination limit), which corresponds to λ = 0.002 for 100 ppm. It can be concluded that at least 98% of V(IV) is removed from Nafion™. After the extraction, the vanadium concentration in the PVDF-based membrane is even lower and below the detection limit (<35 ppm). It can be concluded that nearly 100% of the vanadium is extracted from the PVDF-based membrane. Hence, the extraction procedure is efficient for Nafion™ and similar ionomeric membranes.

### 3.2. Vanadium Speciation in Nafion™ and PVDF-Based Membrane

**Figure 7:**
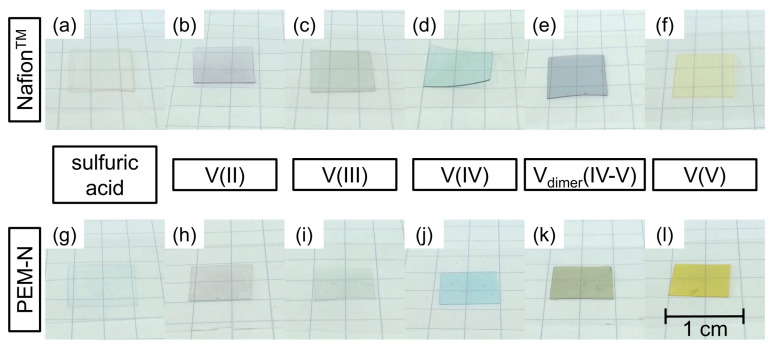
Photographs of Nafion™ hydrated with (**a**) 4 M sulfuric acid and hydrated with 1.6 M vanadium electrolyte (**b**) V(II), (**c**) V(III), (**d**) V(IV), (**e**) V_dimer_(IV–V), and (**f**) V(V), and PVDF-based membrane hydrated with (**g**) 4 M sulfuric acid and hydrated with 1.6 M vanadium electrolyte (**h**) V(II), (**i**) V(III), (**j**) V(IV), (**k**) V_dimer_(IV–V), and (**l**) V(V).

Beside the concentration, the species of vanadium in Nafion™ and PVDF-based membranes were determined. Membranes immersed in 4 M sulfuric acid, V(II), V(III), V(IV), V_dimer_(IV–V), and V(V) electrolyte were subjected to the species analysis. The color of the membrane is a first indicator for the respective species. In Figure 7, photographs of Nafion™ (a)–(f) and PVDF-based membranes (g)–(l) soaked with electrolyte are shown.

Membranes of both types hydrated with sulfuric acid (s. Figure 7a,g) are transparent and show no significant change compared with the appearance of the untreated membrane (not shown here). The color of the membranes immersed in V(II), V(III), V(IV), and V(V) are similar for both types of membranes and comparable with the colors of the vanadium electrolyte and those reported in literature. V(II) electrolyte is violet, V(III) green, V(IV) blue, and V(V) yellow [63,64].

However, the two membranes hydrated with V_dimer_(IV–V) electrolyte are different in color (s. Figure 7e,k). Nafion™ has a dark blueish tone similar to the initial electrolyte, whereas the PVDF-based membrane has a greenish shade. The specific dark blueish color of Nafion™ observed here indicates that the concentrations of V(IV) and V(V) inside Nafion™ are high enough to stabilize the dimer V_dimer_(IV–V) [39]. The equilibrium constant of the formation of the dimer V_dimer_(IV–V) from V(IV) and V(V) (s. Reaction 1) is quite low (K = 0.8 M^−1^). Accordingly, the dimer is only formed and stabilized in solutions with high concentrations [39].

VO_2_^+^(H_2_O)_4_ + VO^2+^(H_2_O)_5_ ⇌ V_2_O_3_^3+^(H_2_O)_8_ + H_2_O (1)

The greenish color of the PVDF-based membrane instead indicates that the equilibrium shifts from V_dimer_(IV–V) to V(IV) and V(V). Consequently, the dimer is less stabilized and partly dissociated. Possibly, the observed color arises from the subtractive mixing of the colors of V(IV) and V(V).

The absorption of the specimens in the UV/VIS range was studied to obtain more detailed information on the species. The UV/VIS spectra of the individual vanadium species solutions including the dimer are well described in the literature [40,41,42,43,44,46,65]. However, UV/VIS characterization of vanadium species inside the membrane has rarely been carried out [52]. Spectra of the vanadium electrolytes with concentrations of 0.8 M, 0.4 M, and 0.16 M are found in the Supplementary materials (s. Figure S1).

**Figure 8:**
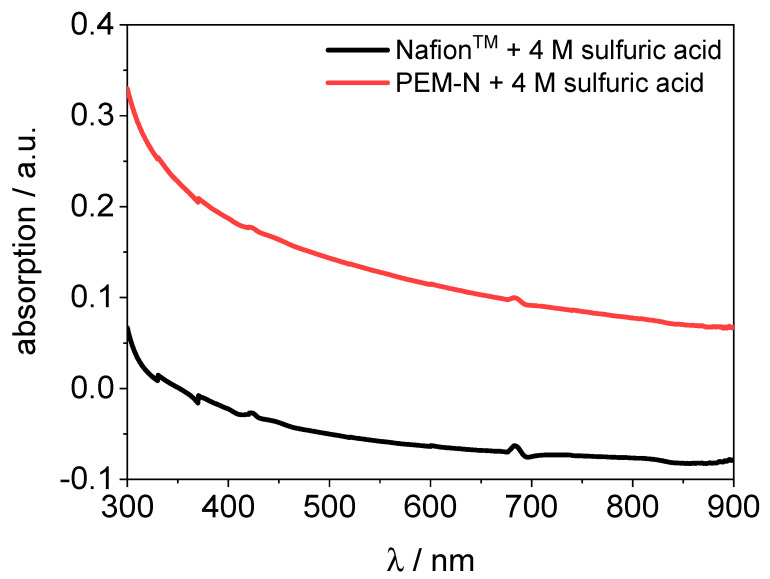
UV/VIS spectra of Nafion™ and a PVDF-based membrane hydrated with 4 M sulfuric acid.

The blank absorption of the PVDF-based membrane is overall three times higher compared to Nafion™. Hence, the absorption of vanadium in the PVDF-based membrane was obtained using a blank membrane (PVDF-based membrane hydrated with 4 M sulfuric acid as a reference). A blank spectrum of Nafion™ and the PVDF-based membrane hydrated with 4 M sulfuric acid is shown in Figure 8.

**Figure 9:**
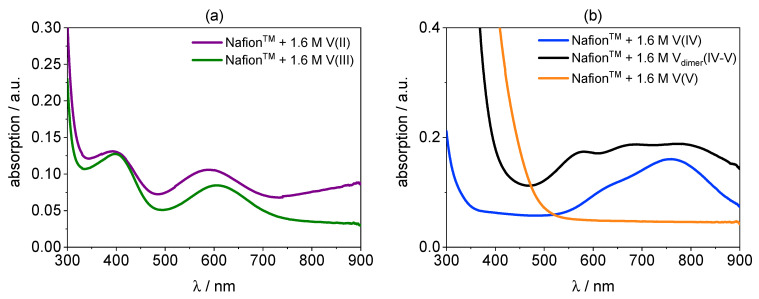
(**a**): UV/VIS spectra of Nafion™ hydrated with V(II) and V(III). (**b**): UV/VIS spectra of Nafion™ hydrated with V(IV), V_dimer_(IV–V), and V(V).

In Figure 9, the UV/VIS spectra of Nafion™ hydrated with V(II), V(III), V(IV), V_dimer_(IV–V), and V(V) electrolyte are shown. The UV/VIS spectra of the Nafion™ membranes soaked with all vanadium species match very well with the spectra of the original electrolytes. The UV/VIS method is applicable for membranes soaked in bathing solutions with concentrations of 0.8 M, 0.4 M, and 0.16 M (s. Figures S2–S6).

**Figure 10:**
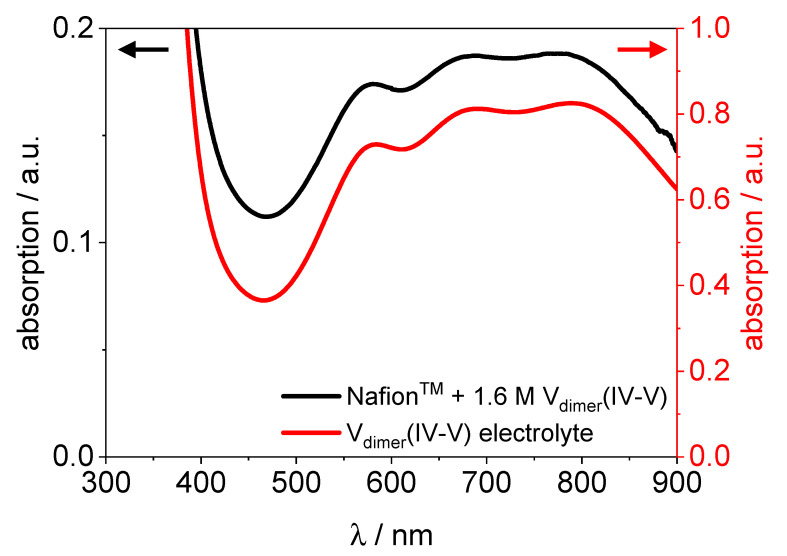
UV/VIS spectra of Nafion™ hydrated with V_dimer_(IV–V) and 1.6 M V_dimer_(IV–V) electrolyte.

The spectrum of V(II) in the membrane shows strong absorption peaks at 400 nm and 590 nm. Additionally, a weak peak at 890 nm is present. The spectrum of V(III) in Nafion™ shows strong peaks at 400 nm and 610 nm. The V(IV) species in the membrane absorbs strongly at 765 nm and at lower wavelengths from 330 nm increasing toward lower wavelength. The spectrum of V(V) is quite plain. It shows just an increase toward lower wavelengths at 520 nm. In Figure 10, the UV/VIS spectra of V_dimer_(IV–V) in Nafion™ and in electrolyte are shown. It is obvious that the spectra are mostly identical, besides the overall intensity of the absorption.

The spectrum of the V_dimer_(IV–V) uptake experiment in Nafion™ shows strong absorption at 575 nm, 680 nm, and 795 nm, just as the dimer spectrum of the electrolyte. Consequently, the results indicate that the V_dimer_(IV–V) dimer diffuses into the membrane. That applies only partly for membranes soaked in bathing solutions with concentrations of 0.8 M, 0.4 M, and 0.16 M (s. Figures S2–S6). The finding suggests that for bathing solutions with a concentration of 0.16 M vanadium, the dimer decays in Nafion™, as well.

**Figure 11:**
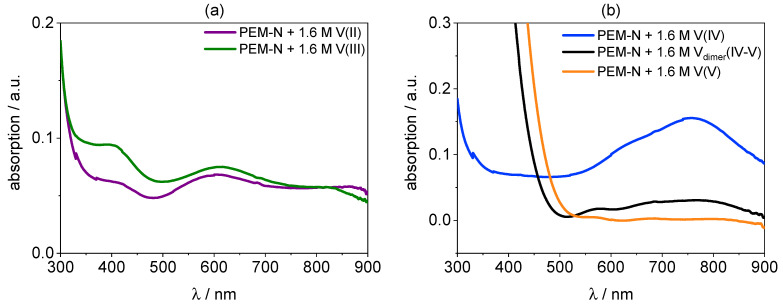
(**a**): UV/VIS spectra of a PVDF-based membrane hydrated with V(II) and V(III). (**b**): UV/VIS spectra of a PVDF-based membrane hydrated with V(IV), V_dimer_(IV–V) (corrected), and V(V) (corrected).

The UV/VIS spectra of a PVDF-based membrane hydrated with V(II), V(III), V(IV), V_dimer_(IV–V), and V(V) electrolyte are presented in Figure 11. The spectrum from the PVDF-based membrane required background fitting and subtraction for V_dimer_(IV–V) and V(V) (s. experimental).

The spectra of the PVDF-based membrane immersed in V(II), V(III), V(IV), and V(V) are similar to the respective Nafion™ spectra and in agreement with the spectra of the electrolyte obtained in this work (s. Figure S1) and reported in literature [44,66].

**Figure 12:**
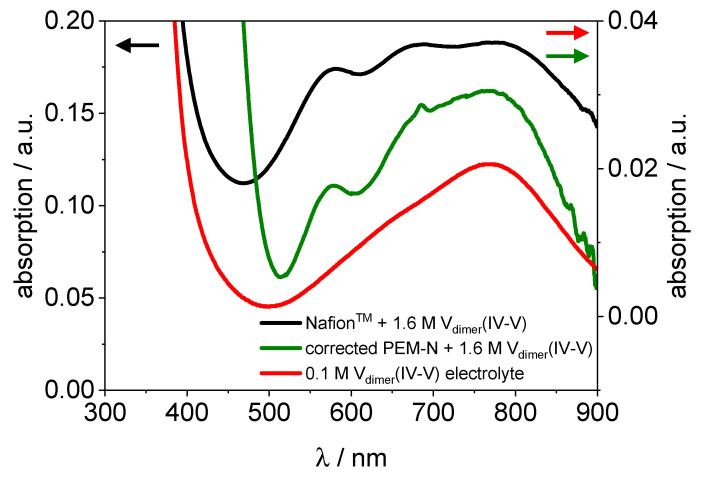
UV/VIS spectra of the PVDF-based membrane hydrated with V_dimer_(IV–V) (corrected), Nafion™ hydrated with V_dimer_(IV–V) and 0.1 M V_dimer_(IV–V) electrolyte.

Most intriguing are the spectra of PVDF-based membrane hydrated with 1.6 M V_dimer_(IV–V) electrolyte, because they are significantly different from those of the dimer in Nafion™ and of the V_dimer_(IV–V) electrolyte (s. Figure 9, Figure 10 and Figure 12). As stated above the V_dimer_(IV–V) spectrum in Nafion™ shows a strong absorption band between 460 nm and 900 nm with three shoulders at 575 nm, 680 nm, and 795 nm similar to the electrolyte (s. Figure 9). Additionally, an increase to lower wavelengths at 450 nm is present. The spectrum of the PVDF-based membrane also shows an absorption band between 500 nm and 900 nm. The absorption is significantly weaker compared to the spectra in Nafion™ and just two shoulders at 570 nm and 680 nm are present.

In Figure 12, the UV/VIS spectra of PVDF-based membrane hydrated with V_dimer_(IV–V), Nafion™ hydrated with V_dimer_(IV–V) and 0.1 M V_dimer_(IV–V) electrolyte are displayed for comparison. The spectrum of the PVDF-based membrane hydrated with V_dimer_(IV–V) is neither comparable with the spectrum of Nafion™ hydrated with V_dimer_(IV–V) nor with the spectrum of 0.1 M V_dimer_(IV–V) electrolyte. However, the strong absorption peak at 575 nm is present, which is specific for V_dimer_(IV–V).

From these results, we conclude that the dimer partly dissociates in the PVDF-based membrane. This shift of the equilibrium is well known from the electrolyte, with decreasing vanadium concentration the dimer V_dimer_(IV–V) decays into V(IV) and V(V) [39]. This is observed when diluting V_dimer_(IV–V) electrolyte from 1.6 M to 0.1 M (Figure S7). The fraction of the respective species could not be determined at this point. The UV/VIS spectrum of the dimer cannot be constructed from a linear combination fit of V(IV) and V(V) spectra because of the very strong absorption compared to the individual species [41,42,44,46]. Instead, experimental approaches have been successfully pursued by Petchsingh et al. [46]:

In an iterative process, they obtained spectra from 50% SOC VRFB PE at different concentrations. They observed, with decreasing vanadium concentrations from 1.6 M to 0.35 M, the spectra of V_dimer_(IV–V) become closer to the V(IV) spectra. The explanation for this observation is that the dimer dissociates with increasing dilution and the spectrum of V(IV) becomes dominant over the others. As discussed above, similar processes are expected to occur in the PVDF-based membrane. In Petchsingh et al., the spectrum of the 0.35 M 50% SOC PE is quite similar to the one observed here (s. Figure 12 green line). When the concentration is decreased further to 0.1 M, the spectra resemble the one of V(IV) even more (s. Figure 12 red line).

Thus, we postulate that the equilibrium of the dimer formation in the PVDF-based membrane shifts to the constituent ions V(IV) and V(V). The UV/VIS results confirm the optical observation, which shows a color difference between Nafion™ and PVDF-based membrane with respect to the vanadium dimer species (s. Figure 7).

**Figure 13:**
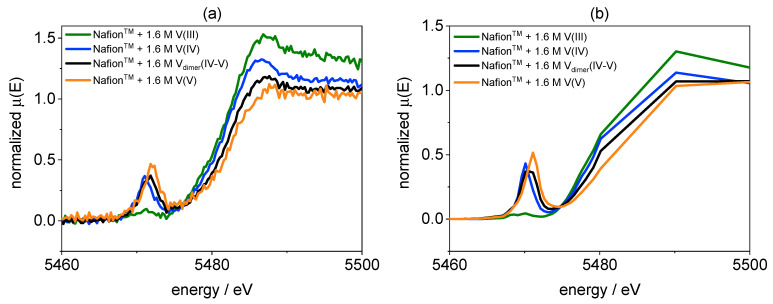
(**a**): V K-edge of Nafion™ hydrated with V(III), V(IV), V_dimer_(IV–V), and V(V) electrolyte performed using the laboratory-based device easyXES100 (n = 10). (**b**): V K-edge of Nafion™ hydrated with V(III), V(IV), V_dimer_(IV–V), and V(V) electrolyte performed at BAM*line* (n = 1).

The UV/VIS information on the vanadium species in the membranes were evaluated using XANES as an independent method. Generally, XANES provides information on the oxidation state of an element and its local coordination. In Figure 13, the V K-edge spectra of V(III), V(IV), V_dimer_(IV–V), and V(V) obtained from Nafion™ hydrated with the respective single element electrolytes are shown. The spectra were measured with a laboratory-based XANES and in addition at the BAMline using a synchrotron source. The spectra are comparable even if the signal-to-noise ratio is significantly lower for the data obtained in the laboratory.

The XANES spectra of V(III) in Figure 13 does not show a pre-edge peak. Due to the octahedral coordination of V(III), the 1s → 3d transition is not allowed. However, distorted octahedral structure of the V(IV), V_dimer_(IV–V), and V(V) species allows for this transition. Thus, the XANES of these ions show a pre-edge peak [67]. Furthermore, the change of the pre-edge peak energy and intensity is specific for V(IV), V_dimer_(IV–V), and V(V). In addition, the edge shifts with increasing formal oxidation state to higher energies. The spectra from PVDF-based membranes are nearly the same as those obtained from Nafion™ (not shown here). The XANES spectra of the different vanadium species also match those published by Jia et al. [51].

Since the spectra for both membranes are similar, we expect a) a similar chemical environment in both membranes and b) V(V) and V(IV) having similar concentrations. We found that XANES is not sensitive for the dimer, but it is possible to determine the ratio of vanadium of V(V) and V(IV) in both membranes. The composition of V_dimer_(IV–V) in Nafion™ is 46% V(V) and 54% V(IV). In comparison, the result for the PVDF-based membrane is 50% V(IV) and 50% V(V). Both membranes have approx. the same vanadium species ratio. However, combined with the visual observation and the UV/VIS results only in Nafion™ the dimer is stabilized. Instead, in the PVDF-based membrane, the single monovalent vanadium species are coexisting.

## 4. Conclusions

We have shown that single vanadium species inside the membrane can be determined by UV/VIS and the spectra are similar to those of the corresponding vanadium electrolyte. Comparing the uptake of V_dimer_(IV–V) in Nafion™ with the PVDF-based membrane, we found that the dimer is stable in Nafion™. In contrast, the dimer is less stabilized in the PVDF-based membrane and thus, the individual V(IV) and V(V) species are coexisting besides the dimer. So far, the transport of V_dimer_(IV–V) was not considered in published models. For a better understanding of the VRFB and vanadium crossover, the transport of V_dimer_(IV–V) should be included in the models. Hence, through a better understanding of the crossover, the capacity loss of VRFB can be minimized and the performance of the battery system can be increased. Especially during the operation of the VRFB, the dimer is present from 10% to 90% SOC. Consequently, it is required to determine the individual diffusion coefficient of V_dimer_(IV–V), as necessary for all other vanadium species.

We compared and evaluated the UV/VIS results with XANES measurements. The XANES spectra of V inside the membranes are similar to those obtained from the electrolyte. XANES measurements validated the UV/VIS results for single/individual species of V(III), V(IV), and V(V). Regarding the dimer, the results obtained by UV/VIS and XANES are complementary. The XANES is not sensitive to the dimeric species but yields a linear combination of V(IV) and V(V) regardless weather if those are bound or they are individual ions. Thus, UV/VIS is a suitable method for the speciation of single vanadium species including the dimer in transparent membranes. Furthermore, XANES is able to determine V(III) and the fraction of V(IV) and V(V) inside the membrane.

Methodically, we presented a novel procedure for the extraction of vanadium ions from membranes and evaluated it using microXRF. Nearly all vanadium ions were extracted from Nafion™ and PVDF-based membranes. Therefore, it was possible to determine the vanadium concentration inside the membranes which was in the range of 2.54 mg·g^−1^ (V(V)) to 7.17 mg·g^−1^ (V(II)). The following order of uptake of vanadium species from single species electrolyte solutions was determined: V(II) > V(III) > V(IV) > V(V) > V_dimer_(IV–V). These data show that the V_dimer_(IV–V) behaves differently from V(IV) and V(V) and needs to be treated individually.

## Data Availability

Not applicable.

## Supplementary Materials

The following are available online at https://www.mdpi.com/article/10.3390/membranes11080576/s1, Figure S1: UV/VIS spectra of 1.6 M V(II), 1.6 M V(III), 1.6 M V(IV), 1.6 M V_dimer_(IV–V), and 1.6 M V(V) electrolyte, Figure S2: UV/VIS spectra of Nafion™ hydrated with 0.8 M, 0.4 M, and 0.16 M V(II) electrolyte, Figure S3: UV/VIS spectra of Nafion™ hydrated with 0.8 M, 0.4 M, and 0.16 M V(III) electrolyte, Figure S4: UV/VIS spectra of Nafion™ hydrated with 0.8 M, 0.4 M, and 0.16 M V(IV) electrolyte, Figure S5: UV/VIS spectra of Nafion™ hydrated with 0.8 M, 0.4 M, and 0.16 M V_dimer_(IV–V) electrolyte, Figure S6: UV/VIS spectra of Nafion™ hydrated with 0.8 M, 0.4 M, and 0.16 M V(V) electrolyte, Figure S7: Photograph of V_dimer_(IV–V) electrolyte with the concentrations 1.6 M (a) and 0.1 M (b).
