# Supplementary material for: Characterization of Dimeric Vanadium Uptake and Species in Nafion™ and Novel Membranes from Vanadium Redox Flow Batteries Electrolytes
Source: Membranes (Basel). 2021 Jul 29;11(8):576. doi: 10.3390/membranes11080576 (PMC8399489; doi:10.3390/membranes11080576)
Supplement: Supplementary file 1 [file membranes-11-00576-s001.zip › membranes-1317173-supplementary.pdf]

membranes

Supplementary Materials:

Characterization of Dimeric Vanadium Uptake and Species in Nafion™ and Novel Membranes from Vanadium Redox Flow Batteries Electrolytes

Christian Lutz [1], Michael Breuckmann [2], Sven Hampel [1], Martin Kreyenschmidt [2], Xi Ke [3], Sabine Beuermann [3], Katharina Schafner [4,5], Thomas Turek [4,5], Ulrich Kunz [4,5], Ana Guilherme Buzanich [6], Martin Radtke [6] and Ursula E. A. Fittschen [1,*]

[1] Clausthal University of Technology, Institute of Inorganic and Analytical Chemistry, Arnold-Sommerfeld-Str. 4, 38678 Clausthal-Zellerfeld, Germany; christian.lutz@tu-clausthal.de (C.L.), sven.hampel@tu-clausthal.de (S.H.)
[2] University of Applied Science Münster, Department of Chemical Engineering, Stegerwaldstr. 39, 48565 Steinfurt, Germany; michael.breuckmann@fh-muenster.de (M.B.), martin.kreyenschmidt@fh-muenster.de (M.K.)
[3] Clausthal University of Technology, Institute of Technical Chemistry, Arnold-Sommerfeld-Str. 4, 38678 Clausthal-Zellerfeld, Germany; xi.ke@outlook.de (X.K.), sabine.beuermann@tu-clausthal.de (S.B.)
[4] Clausthal University of Technology, Institute of Chemical and Electrochemical Process Engineering, Leibnizstr. 17, 38678 Clausthal-Zellerfeld, Germany
[5] Forschungszentrum Energiespeichertechnologien, Am Stollen 19A, 38640 Goslar, Germany; katharina.schafner@alumni.tu-clausthal.de (K.S.); turek@icvt.tu-clausthal.de (T.T.); kunz@icvt.tu-clausthal.de (U.K.)
[6] Federal Institute for Materials Research and Testing (BAM), Richard-Willstaetter-Str. 11, 12489 Berlin, Germany; ana.buzanich@bam.de (A.G.B.); martin.radtke@bam.de (M.R.)
* Correspondence: ursula.fittschen@tu-clausthal.de (U.E.A.F.); Tel.: +49 (0) 5323 72 2205

In Figure S1, the UV/VIS spectra of 1.6 M V(II), 1.6 M V(III), 1.6 M V(IV), 1.6 M V(IV/V), and 1.6 M V(V) electrolyte are shown. The V(II) and V(III) electrolyte were analyzed using a 1 mm cuvette and the V(IV), V(IV/V), and V(V) electrolyte using a 0.1 mm flow through cuvette. The data are in agreement with the literature [1,2].

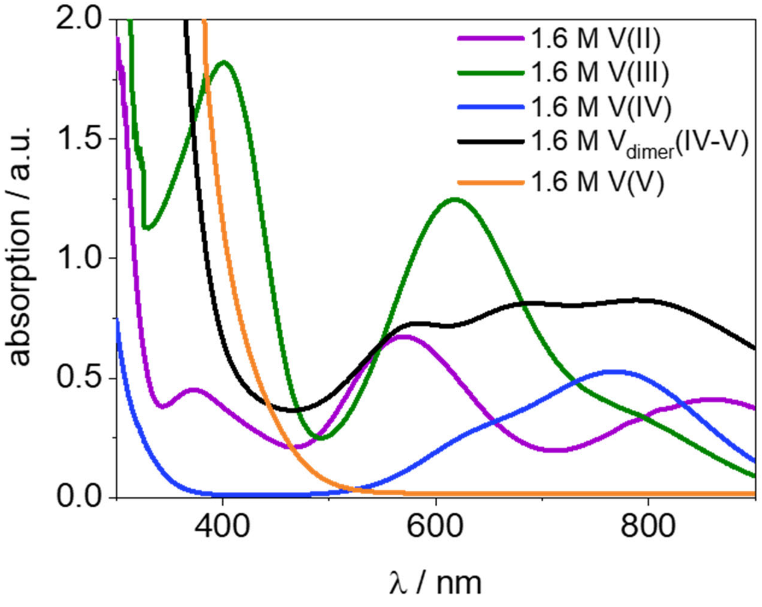

Figure S1. UV/VIS spectra of 1.6 M V(II), 1.6 M V(III), 1.6 M V(IV), 1.6 M V(IV/V), and 1.6 M V(V) electrolyte.

In Figure S2 to S6, UV/VIS spectra of Nafion™ hydrated with either V(II), V(III), V(IV), V(IV/V), and V(V) electrolyte with the concentrations 0.8 M, 0.4 M and 0.16 M are shown. As discussed in the result section, UV/VIS spectra of Nafion™ hydrated with lower concentrations of vanadium provide good data for the speciation.

The same measurements were applied to the PVDF-based membrane. It was evident that the PVDF-based membrane hydrated in 0.8 M vanadium electrolyte provides not evaluable spectra anymore. Considering the uptake of the PVDF-based membrane compared to Nafion™ must be significantly lower.

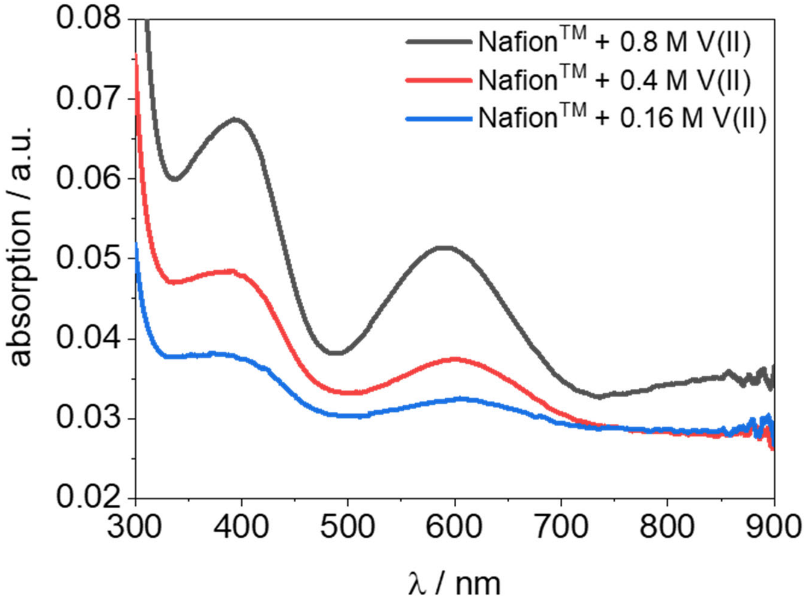

Figure S2. UV/VIS spectra of Nafion™ hydrated with 0.8 M, 0.4 M, and 0.16 M V(II) electrolyte.

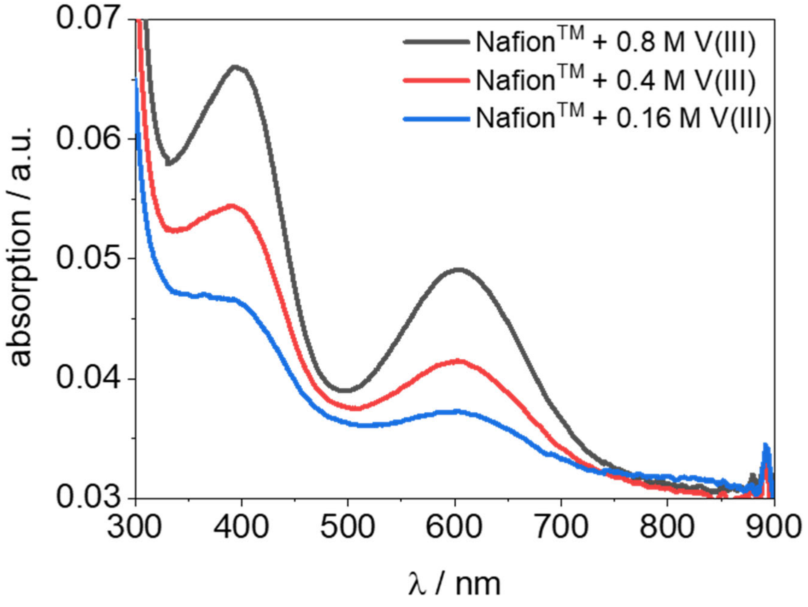

Figure S3. UV/VIS spectra of Nafion™ hydrated with 0.8 M, 0.4 M, and 0.16 M V(III) electrolyte.

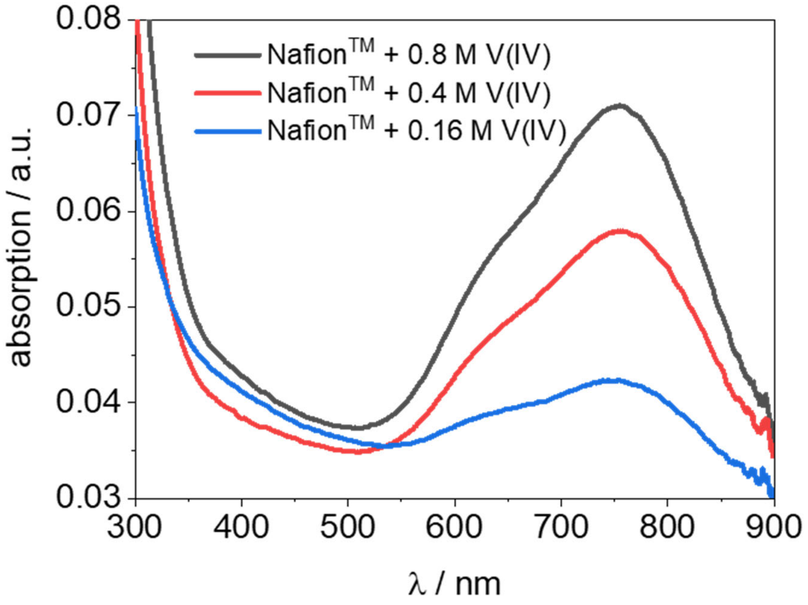

Figure S4. UV/VIS spectra of Nafion™ hydrated with 0.8 M, 0.4 M, and 0.16 M V(IV) electrolyte.

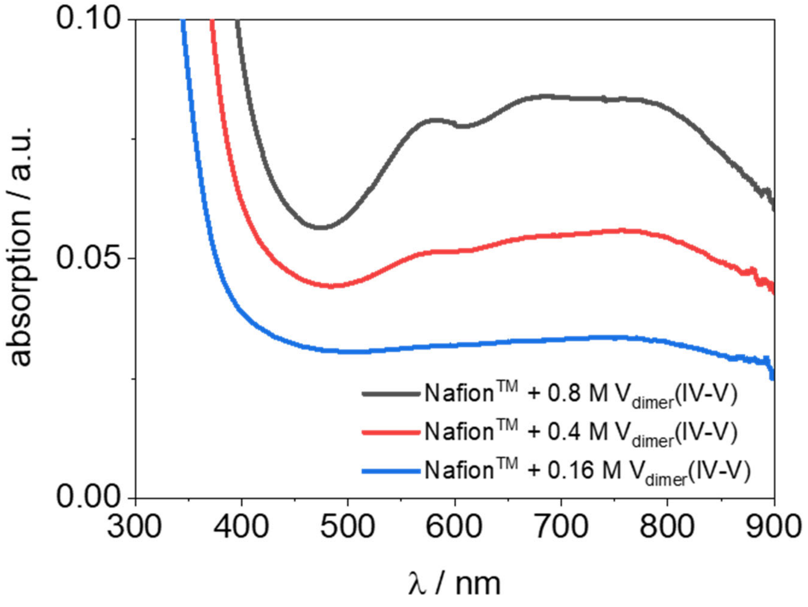

Figure S5. UV/VIS spectra of Nafion™ hydrated with 0.8 M, 0.4 M, and 0.16 M V(IV/V) electrolyte.

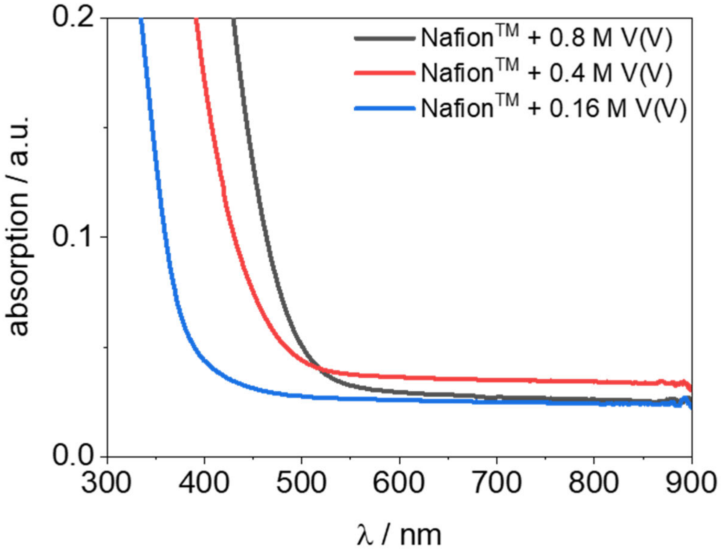

Figure S6. UV/VIS spectra of Nafion™ hydrated with 0.8 M, 0.4 M, and 0.16 M V(V) electrolyte.

In Figure S7, a photograph of V(IV/V) electrolyte is shown with the concentrations 1.6 M (a) and 0.1 M (b).

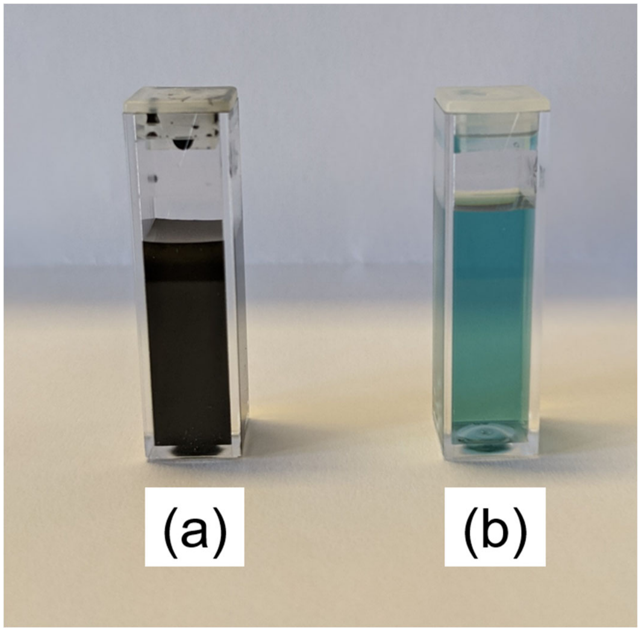

Figure S7. Photograph of V(IV/V) electrolyte with the concentrations 1.6 M (a) and 0.1 M (b).

References

1. Buckley, D.N.; Gao, X.; Lynch, R.P.; Quill, N.; Leahy, M.J. Towards Optical Monitoring of Vanadium Redox Flow Batteries (VRFBs): An Investigation of the Underlying Spectroscopy. *J. Electrochem. Soc.* **2014**, *161*, A524–A534, doi:10.1149/2.023404jes.

2. Petchsingh, C.; Quill, N.; Joyce, J.T.; Eidhin, D.N.; Oboroceanu, D.; Lenihan, C.; Gao, X.; Lynch, R.P.; Buckley, D.N. Spectroscopic Measurement of State of Charge in Vanadium Flow Batteries with an Analytical Model of V^{IV}-V^{V} Absorbance. *J. Electrochem. Soc.* **2016**, *163*, A5068–A5083, doi:10.1149/2.0091601jes.
